# Particle-in-cell simulation of the effect of dust charge fluctuation on ion acoustic waves in a dusty plasma

**DOI:** 10.1038/s41598-020-77772-x

**Published:** 2020-12-01

**Authors:** Suniti Changmai, Madhurjya P. Bora

**Affiliations:** Physics Department, Gauhati University, Guwahati, 781014 India

**Keywords:** Fluid dynamics, Plasma physics

## Abstract

A new hybrid-particle-in-cell (PIC)-Monte Carlo Collision (h-PIC-MCC) algorithm is presented here. The code correctly simulates the damping of ion acoustic wave due to dust charge fluctuation in a dusty plasma along with other kinetic effects such as Landau damping. In the model, on event of a collision between a charged particle and a dust particle, a randomised probability determines whether the charged particle is absorbed by the dust with the collision cross section being determined dynamically by the overall interaction scenario. We find that this method is versatile enough as it can also include the size and mass distribution for the dust particles, in addition to the charged species dynamics. As such, it can be adopted to study numerous phenomena that occur in diverse dusty plasma environments. We believe that the damping of the ion acoustic wave through dust charge fluctuation is being demonstrated, for the first time, with a PIC code, in this work.

## Introduction

Dusty plasma provides a broad environment for carrying out extensive research in the field of plasma physics. Examples of dusty plasma can range widely, from laboratory to space plasma environments^1–3^. Such a system is characterised by the presence of dust particles whose radius can vary from nanometers to micrometers in size. The fundamental question that arises in any dusty plasma environment is how these particles charge when immersed in plasma. Thus, the dust charging process has always been a subject of wide interest^4–6^. As a consequence of the dust charging process, numerous phenomena can occur in the plasma. A significant outcome among them is the exhibition of self-consistent dust charge fluctuation that results as a response to the variation in the background plasma currents into the dust particles^5,7–9^. This fluctuation can lead to the damping of the plasma waves as well as development of other instabilities^10–15^. We consider the effect of such dust charge fluctuation on the electrostatic ion acoustic wave as the primary subject of this investigation. Some notable early contributions in this field include the works of Jana et al.^10^ and Ma et al.^12^, which consider the damping of ion acoustic wave due to dust charge fluctuation, using fluid and kinetic theory, respectively. In this work, we perform a kinetic particle-in-cell (PIC) simulation to study the characteristics of the ion acoustic wave in presence of dust charge fluctuation in a dusty plasma. Following the general outline^16^, we adopt a modified PIC algorithm to estimate the collisions of the dusts with the electrons and ions. We thus use our hybrid-PIC-Monte Carlo Collision (*h-PIC-MCC)* method to estimate the damping of the dust-ion-acoustic (DIA) wave, which we believe, has been carried out for the first time with a PIC simulation. We find that our numerical results corroborate well with the theoretical approximations.

In this work, the primary emphasis is on the effect of dust charge fluctuation on the ion acoustic wave rather than the detailed dust charging process. As we shall indicate in the relevant sections, our numerical scheme does compromise on the details of the dust charging process at the expense of gaining speed in the simulation. We show that despite this shortcoming, the simulation model correctly captures all the important signatures of the effect of dust charge and its dynamic behaviour on ion acoustic wave. Throughout our work, in many places we refer to the dust-ion-acoustic (DIA) wave as ion acoustic wave (IA) as the dust particles remain stationary and dynamically the dust dynamics does not enter into the ion acoustic oscillations. In “The theory of dust-ion-acoustic wave” section, we briefly describe the fluid and kinetic theories of DIA wave. In “Simulation model” section, we describe our simulation model in detail. The simulation results are described in “Simulation results” section. We conclude our work in “Conclusions” section. An “Appendix” outlining the computational details can be found at the end.

## The theory of dust-ion-acoustic wave

We first consider the excitation of electrostatic ion acoustic wave in an *e*-*i* plasma without either resorting to plasma approximation or Boltzmannian electrons. The model is described by the electron and ion continuity and momentum equations,

1 $$\begin{aligned} \frac{\partial n_{j}}{\partial t}+\nabla .(n_{j}{\varvec{v}}_{j})=  0, \end{aligned}$$

2 $$\begin{aligned} m_{j}n_{j}\frac{d{\varvec{v}}_{j}}{dt}= & {} q_{j}n_{j}{\varvec{E}}-\nabla p_{j}, \end{aligned}$$

where $j=e,i$ for electrons and ions respectively, $q_{i,e}=\pm e$ is the electronic charge, $p_{j}=\gamma _{j}n_{j}T_{j}$ are thermal pressures and other symbols have their usual meanings. We have expressed the temperature in the energy unit. The equations are closed by the Poisson equation

3 $$\begin{aligned} \epsilon _{0}\nabla \cdot {\varvec{E}}=e(n_{i}-n_{e}). \end{aligned}$$

The linear dispersion relation of this fluid model can be written as^17,18^,

4 $$\begin{aligned} 1-\frac{\omega _{pi}^{2}}{\omega ^{2}-\gamma _{i}k^{2}v_{\mathrm{th}i}^{2}}-\frac{\omega _{pe}^{2}}{\omega ^{2}-\gamma _{e}k^{2}v_{\mathrm{th}e}^{2}}=0, \end{aligned}$$

with $\omega _{pj}=\sqrt{n_{0}e^{2}/(\epsilon _{0}m_{j})}$ are the respective plasma frequencies and $v_{\mathrm{th}j}=\sqrt{T_{j}/m_{j}}$ are the thermal velocities, where $n_{0}=n_{i0}\simeq n_{e0}$ is the equilibrium plasma density. The above dispersion relation is being used to benchmark our simulation.

In what follows, we briefly discuss the kinetic theory behind the dust charge fluctuation dynamics and related kinetic effects^9,12^. We consider the 1-D linear ion acoustic wave with a characteristic frequency $\omega$ lying in the range $\omega _{pd}\ll \omega \ll kv_{\mathrm{th}e}$, where $\omega _{pd}$ is the dust plasma frequency. The kinetic theory of ion acoustic wave with dust charge dynamics is due to the Boltzmann equation

5 $$\begin{aligned} \frac{\partial f_{j}}{\partial t}+{\varvec{v}}_{j}\cdot \nabla f_{j}-\frac{q_{j}}{m_{j}}\nabla \phi \cdot \nabla _{v}f_{j}=\mathcal{F}_{\mathrm{coll}}, \end{aligned}$$

where $f_{j}\equiv f_{i,e}$ are the ion and electron distribution functions, $\phi$ is the electrostatic potential, and $\mathcal{F}_{\mathrm{coll}}$ is the collision operator which represents the rate of electron and ion capture by the dust particles thus incorporating the dust charge dynamics. In the linear theory, for negatively charged dust particles, which is the case of this present work, the oscillating current to the dust grain is given by

6 $$\begin{aligned} \delta I=\eta Q_{d1}+\sum _{j=i,e}q_{j}\int _{v_{mj}}^{\infty }v_{j}\sigma _{j} (v_{j},Q_{d0})f_{j1}\,d^{3}v_{j}, \end{aligned}$$

where $\sigma _{j}$ is the effective collision cross section of the respective species with the dust particles and $\eta$ is the charge relaxation rate

7 $$\begin{aligned} \eta =\frac{e|I_{e0}|}{C}\left( \frac{1}{T_{e}}+\frac{1}{T_{i}-eQ_{d0}/C}\right) , \end{aligned}$$

$Q_{d0,d1}$ are the dust charges, and *C* is the capacitance of a dust particle. The lower limit of the integration $v_{mj}=v_{m},0$, respectively for electrons and ions, is the minimum velocity with which the electron or the ion must reach the surface of the dust particle. In the above expression and in the expressions hereafter, the subscript ‘0’ and ‘1’ refer to the equilibrium and perturbed values of the respective quantities. The expression for the collision cross section is

8 $$\begin{aligned} \sigma _{j}(v_{j},Q_{d0})=\pi r_{d}^{2}\left( 1-\frac{2q_{j}Q_{d0}}{Cm_{j}v_{j}^{2}}\right) , \end{aligned}$$

where $r_{d}$ is the average dust radius. The equations are closed by the linearised Poisson equation

9 $$\begin{aligned} \epsilon _{0}\nabla ^{2}\phi _{1}=en_{e1}-en_{i1}-Q_{d0}n_{d1}-Q_{d1}n_{d0}. \end{aligned}$$

The linear dispersion relation for ion acoustic wave can be written as

10 $$\begin{aligned} 1+\chi _{e}+\chi _{i}+\frac{i}{\omega +i\eta }\left( \frac{\eta _{e}}{k^{2}\lambda _{\mathrm{D}e}^{2}}-\eta _{i}\frac{\omega _{pi}^{2}}{\omega ^{2}}\right) =0, \end{aligned}$$

where

11 $$\begin{aligned} \chi _{e}= & {} \frac{1}{k^{2}\lambda _{\mathrm{D}e}^{2}}\left( 1 +i\frac{\omega }{kv_{\mathrm{th}e}}\sqrt{\frac{\pi }{2}}\right) , \end{aligned}$$

12 $$\begin{aligned} \chi _{i}= & {} -\frac{\omega _{pi}^{2}}{\omega ^{2}}\left( 1+\frac{3k^{2}v_{\mathrm{th}i}^{2}}{\omega ^{2}}\right) +i\chi _{m}, \end{aligned}$$

13 $$\begin{aligned} \chi _{m}= & {} \frac{\omega }{k^{3}\lambda _{\mathrm{D}i}^{2}v_{\mathrm{th}i}}\sqrt{\frac{\pi }{2}}\exp \left( -\frac{\omega ^{2}}{2k^{2}v_{\mathrm{th}i}^{2}}\right) , \end{aligned}$$

14 $$\begin{aligned} \eta _{e}= & {} \delta _{e}\frac{|I_{e0}|}{e}, \end{aligned}$$

15 $$\begin{aligned} \eta _{i}= & {} -\delta _{i}\frac{\omega ^{2}}{k^{2}}\int _{0}^{\infty }\frac{\partial f_{i0}/\partial v_{x}}{\omega /k-v_{x}}v_{i}\sigma _{i}(v_{i},Q_{d0})\,d^{3}v_{i}, \end{aligned}$$

where $\delta _{e,i}$ are the ratios of equilibrium dust density to that of electron and ion densities. In the above expressions, $\chi _{e,i}$ are the dielectric susceptibilities and $\eta _{e,i}$ represent the coupling of dust charge fluctuation with electron and ion density perturbations. Eq. (10) predicts damping of the ion acoustic wave, which is due to the dust charge fluctuation. Besides, $\eta _{i}$ also contains the Landau damping term for the wave.

We also note that, even without dust charge fluctuation, the ion acoustic wave is subjected to Landau damping. By neglecting the dust charge fluctuation in the above analysis, the Landau damping rate $\gamma _{L}$ for ion acoustic wave can be approximated as^9^

16 $$\begin{aligned} \gamma _{L}\simeq \sqrt{\frac{\pi }{8}}\,\frac{kc_{s}}{\left( 1+k^{2}\lambda _{\mathrm{D}e}^{2}\right) ^{2}}\left[ \left( \delta _{n}^{-1} \frac{m_{e}}{m_{i}}\right) ^{1/2}+\left( \delta _{n}^{-1} \frac{T_{e}}{T_{i}}\right) ^{3/2}e^{-\zeta -3/2}\right] , \end{aligned}$$

where $\delta _{n}=n_{e0}/n_{i0}$ is the ratio of equilibrium electron to ion densities, $c_{s}=\omega _{pi}\lambda _{\mathrm{D}e}$, and

17 $$\begin{aligned} \zeta =\frac{T_{e}}{T_{i}}\,\frac{1}{2\delta _{n}\left( 1+k^{2}\lambda _{\mathrm{D}e}^{2}\right) }. \end{aligned}$$

**Figure 1:**
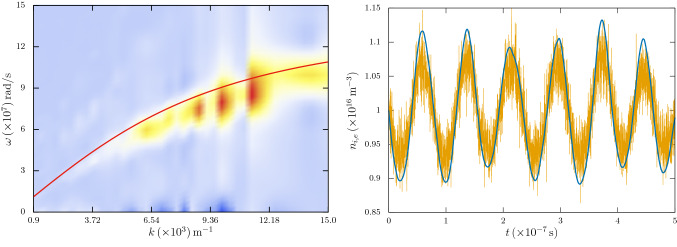
(left panel) The spectral plot of the ion acoustic dispersion (without any dust) relation from the simulation. The solid curve is the theoretically calculated one. (right panel) The corresponding ion acoustic wave in electron and ion densities $n_{i,e}$. The solid line is the ion density and the other one is electron density fluctuation. (Generated using Gnuplot, Version 5.2 patchlevel 7, URL : http://www.gnuplot.info).

## Simulation model

Our primary simulation model comprises of a 1-D electrostatic *e*-*i* dusty plasma with periodic boundary conditions. The dynamics is controlled with an electrostatic particle-in-cell (PIC) code. Both electrons and ions are considered as thermal particles, distributed with their respective distributions, while the dust particles are assumed to be cold. The simulation parameters correspond to a typical laboratory situation with plasma density ${n}_{{0}}\sim {10^{16}}\,\mathrm{m}^{-3}$ and dust density of $\sim 10^{11}\,\mathrm{m^{-3}}$. The electron and ion temperatures are respectively $T_{e}\sim 1\,\mathrm{eV}$ and $T_{i}\sim 0.01\,\mathrm{eV}$, and an *e*-*i* mass ratio of $(m_{e}/m_{i})^{-1}\sim 1835.16$. In a simulation box length of $0.004\,\mathrm{m}$, both electrons and ions are represented with $10^{5}$ macro-particles, while the dust particles are represented with 2500 macro-particles. We carry out our simulation with 600 equally spaced 1-D cells. The electron Debye length of the plasma is $\lambda _{\mathrm{D}e}\sim 7.4\times 10^{-5}\,\mathrm{m}$ and the evolution time step is $\sim 10^{-11}\,\mathrm{s}$. With these simulation parameters, we are able to have full spatial resolution with a temporal resolution of the order of the electron time scale. In Fig. 1 (left panel), we have shown the spectral plot of the ion acoustic dispersion relation in absence of dust particles as determined from the simulation, superimposed with the theoretically calculated curve. The oscillations in Fig. 1 (right panel) show the electron and ion density $(n_{i,e})$ oscillations. While the solid curve represents oscillations in $n_{i}$, the curve with fine-scale oscillations is that in $n_{e}$, where the fine-scale oscillations are due to Langmuir (electron plasma) oscillations modulated by the ion acoustic oscillations. It can be seen that the simulation results are in good agreement with the theory. It is also seen from these curves that the ion density is closely followed by electron density thereby validating the use of plasma approximation $n_{i}\sim n_{e}$ (not used here though).

A few clarifications about our simulation model is in order here. As mentioned at the beginning, the goal of this work is to demonstrate the proof of principle about the effect of dust charge fluctuation on ion acoustic waves in an *e*-*i* plasma, implemented in a PIC code. Toward this, we have used the simplest possible plasma model, namely a fully ionised *e*-*i* hydrogen plasma with stationary dust particles. Various plasma parameters are such that which can be readily implemented in a laboratory^19–22^. We note that though a hydrogen plasma is not the very usual choice for laboratory plasmas, there are laboratory experiments^19,20^ which use hydrogen plasmas. Computationally, as we work in the ion acoustic domain, simulating an ion acoustic wave with heavier ions such as in argon or potassium plasmas and hence longer wavelengths, becomes more challenging. Nevertheless, our computational model, in principle, can be extended to any such laboratory plasma situations. A brief description of the computational model is given in the “Appendix”. In our model, we have neglected the neutrals in the plasma. The presence of neutrals in an *e*-*i* plasma affects the plasma dynamics through neutral collisions. However, as we are not considering any neutral collision here and focus our attention mainly on electrostatic interactions, the neutrals can be neglected as far as the ion acoustic wave is concerned.

### Dust charging with PIC simulation

We now describe our dust-PIC model, which is used to study the dust charging mechanism and its effect in a dusty plasma. It is worth noting that the classical PIC model does not account for collisions^16^. Besides every particle in a PIC model is actually a macro-particle comprising of a number of real-life particles. As such, any collision implemented under the PIC formalism will actually account for collisions en masse. However, there are ways to include collisions among participating particles in PIC, one of which is the popular PIC-Monte Carlo Collision (PIC-MCC) algorithm^23^, usually used for neutral collisions. It consists of using a randomised probability to account for the collisions based on the theoretical estimation of the collision cross sections. Multistep Monte Carlo Collision is another algorithm adopted by Gatsonis et al.^24^ which is based on an algorithm similar to the above but considers dust collisions with the charged species. We however use here a different method to estimate for the collisions of dust particles with electrons and ions, which can be described as the hybrid-PIC-MCC (h-PIC-MCC) method.

#### The hybrid-PIC-MCC scheme

Our hybrid-PIC-MCC (h-PIC-MCC) scheme is based on the Monte Carlo (MC) scheme described by Birdsall^23^. Consider the collision frequency between a dust and a charged particle ‘*s*’ (either an electron or an ion) $\nu _{s}$,

18 $$\begin{aligned} \nu _{s}=n_{d}\sigma _{\mathrm{ab}}v_{s}, \end{aligned}$$

where $n_{d}$ is the local density of dust particles in the particular computational cell. The absorption cross section is $\sigma _{\mathrm{ab}}$ in the event of a collision between a dust particle and a charged particle ‘*s*’ and $v_{s}$ is the relative velocity between the charged particle and the dust particle. However, as in our case the dust particles are stationary, $v_{s}$ is the velocity of the incoming particle. The absorption cross section is given by,

19 $$\begin{aligned} \sigma _{\mathrm{ab}}=\pi r_{d}^{2}\left( 1-\frac{2q_{s}\varDelta \phi }{m_{s}v_{s}^{2}}\right) , \end{aligned}$$

where $\varDelta \phi$ is the potential difference between the dust particle and the local plasma and $r_{d}$ is the radius of the dust particle, and $m_{s}$ and $q_{s}$ are the charge and mass of the colliding charged particle. So, the probability of collision of a charged particle ‘*s*’ within a time interval $\varDelta t$ is given by^23^,

20 $$\begin{aligned} P_{s}=1-e^{-\nu _{s}\varDelta t}. \end{aligned}$$

Note that the above relation for a single species of physical particle can be equivalently written for a computational particle

21 $$\begin{aligned} P^{p}=1-e^{-\nu ^{p}\varDelta t}, \end{aligned}$$

as with sufficient number of collisions, the resultant velocity distribution will resemble that of a single physical particle^23^. We now introduce a uniform random number $0\le \mathcal{R}\le 1$^23,24^ and assume that if $P^{p}>\mathcal{R}$, an absorption occurs, otherwise not. It should be noted that this scheme allows only one collision per time step for a charged particle where either the charged particle is absorbed or remains in the bulk plasma, unlike the multistep MCC process^24^. In a multistep MCC process, the dust particle is treated as the incoming particle which collides with a target particle—a charged particle. Once there is a collision, it checks for more collisions within the time interval over which the system evolves. In both the cases, when an absorption occurs, the computational particle is removed from the bulk plasma and an equivalent charge number is updated for the dust particle accordingly.

However unlike typical PIC-MCC process^23^, we estimate $\nu ^{p}$ through the Coulomb interactions at the grid, which accumulates the charges at the grid points from actual positions of the charged particles through an interpolation process (see “Appendix”). So, instead of estimating the actual absorption cross section for a single dust particle, the process estimates a combined cross section for all the dust particles which are there in a particular cell. We then determine which of the dust particles in a cell would actually absorb the charged particle, depending on the final position of the charged particle and position of the dust particle in that cell. This process is repeated for all the computational particles in the system. This way, we can considerably speed up the calculation of cross section in each time step at the cost of loosing certain charging information for each dust particle which are there in one cell. However, on average the process correctly estimates the dust charge accumulation averaged over a computational cell. In order to account for the dust charge fluctuation in our simulation, we assume that whenever a collision of the dust particle with an electron occurs, it contributes to an increase of negative charge on the surface of the dust particle. This is effected by decreasing the number of electrons in the simulation domain accompanied by an equivalent increase of electron dust charge number $z_{d}$. The accumulation of positive charge on the surface of a dust particle is, however, modeled by assuming that whenever an ion-collision occurs, an electron is ejected from the dust particle which causes $z_{d}$ to decrease (equivalently charging the dust particle positively) and an increase of a plasma electron in the simulation box^8^. Hence, the total ion number in the simulation domain remains constant while the total electron number and dust charge fluctuate depending on the type of collisions.

We note that all the above simplifications in our h-PIC-MCC makes it impossible for this scheme to exactly determine the charging history of individual dust particles while capturing the charging and charge fluctuation dynamics averaged over a computational cell in an ion acoustic time scale. As a result, we can expect the effect of dust charge fluctuation on ion acoustic wave (or dust-ion-acoustic) to be correctly estimated while compromising on the charge history of individual dust particles. This can be considered as a bargain as our emphasis is primarily on the effect of dust charge fluctuation on ion acoustic wave, rather than the charging dynamics of the dust particles.

#### Dust particle versus dust site

As the dust particles are considered stationary, the only way they contribute to the dynamics of the *e*-*i* plasma is through electrostatic interaction. As such the mass of the dust particle as well as their size do not involve in the dynamics of the plasma except for estimating the absorption cross section, which is measured in terms of $r_{d}$. Besides, as far as the electrostatic interaction of the plasma particles with dust particles are concerned, each dust particle behaves like a collection of electrons (as they are negatively charged) with zero temperature (hence zero velocity). Which is why, we would like to refer them as dust sites rather than dust particles. So a dust site with $z_{d}$ number of charges on it behaves as $z_{d}$ number of electrons, occupying the same position in the phase space. In the PIC framework, this is perfectly admissible, as each computational particle can be considered as point particle (compared to the scale length of the plasma) and they can overlap each other^25^.

## Simulation results

### Dust charging

As mentioned in the previous section, we consider the dust component of the plasma to be cold and stationary. The plasma quasineutrality condition^9^ is maintained at all time,

22 $$\begin{aligned} n_{i}=n_{e}+z_{d}n_{d}, \end{aligned}$$

where $n_{i,e,d}$ are the instantaneous physical densities of the ions, electrons, and dust particles. Computationally, the above relation can be represented as

**Figure 2:**
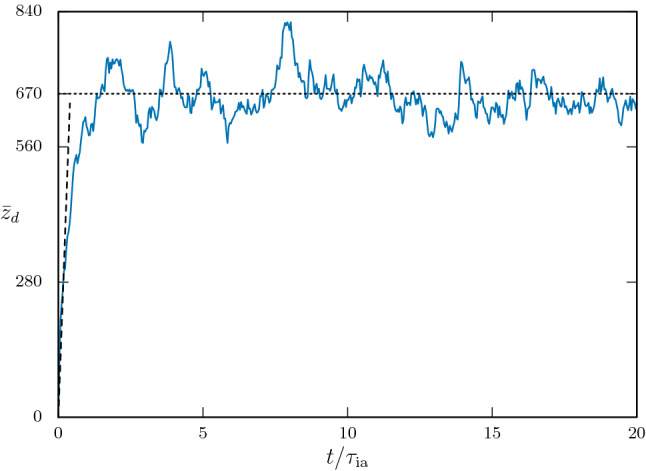
The charging of a single dust particle. ${\bar{z}}_{d}$ is the average dust charge number for a single dust particle. The time is in the unit of ion acoustic time scale $\tau _{\mathrm{ia}}=\omega _{\mathrm{ia}}^{-1}$. (Generated using Gnuplot, Version 5.2 patchlevel 7, URL : http://www.gnuplot.info).

$$\begin{aligned} N^{i}w_{i}=N^{e}(t)w_{e}+{\bar{z}}_{d}(t)N^{d}w_{d}, \end{aligned}$$

where ${\bar{z}}_{d}(t)$ is the instantaneous dust charge number averaged over all the $N^{d}$ computational particles. The quantities $w_{s}=L(n_{s0}/N^{s})$ for each species are called weights, where *L* is the simulation domain length, $n_{s0}$ is the initial physical density for the species ‘*s*’ (at the beginning of the simulation), and $N^{s}(t)$ is the total number of computational particles for the species ‘*s*’ at any instant of time *t*. The weight $w_{s}$ is a dimensionless quantity which measures number of physical (actual) particles of a species that are being represented in a single macro-particle (or computational particle) of the species. So, $N^{s}(t)w_{s}$ is the total number of actual physical particles present in the simulation domain at a time *t*. Note that as per our scheme, $N^{i}(t)\equiv N^{i}=\mathrm{const}.$ and $N^{d}$ are the number of dust sites (or macro-particles) which are fixed in space and time. We use 2500 dust sites, distributed uniformly in the domain with zero net charge. For calculation of the absorption cross section $\sigma _{\mathrm{ab}}$, the dust radius is fixed at $r_{d}\sim 10^{-2}\lambda _{\mathrm{D}e}$. The number of dust sites are chosen so as to have no overlapping of the sites as they occupy the simulation domain. The dust sites can be considered to be equivalent to dust macro-particles, each having radius $r_{d}$. We note here that each computational particle (or macro-particle) consists of a large number of physical particles determined by the weight factor *w* and each computational particle has its shape function $S_{x}$ (see the “Appendix”), which in our case is $S_{x}(x-x^{p})=\delta (x-x^{p})$. Or in other words, the computational particles can still be considered as point particles, so long as the scale length of interactions among themselves is much larger than their size. In our case, this is easily satisfied as our interaction scale length is $\sim \lambda _{De}$ and $r_{d}\ll \lambda _{De}$. Physically, each dust site behaves as a collection of stationary electrons (assuming the dust particles to be negatively charged). We can calculate the equivalent dust charge number ${\bar{z}}_{d}$ by considering the charging current for the dust sites. An orbit motion limited (OML) estimate of the charging current in a dusty plasma is given by^1^

23 $$\begin{aligned} I_{d}=\frac{Q_{d}}{\tau }, \end{aligned}$$

where $\tau$ is the charging time and $Q_{d}=e{\bar{z}}_{d}$ is the average dust charge. Computationally we can only find out the depletion of electron macro-particles in each time step as they get absorbed in the dust sites, which provides the total current $I_{\mathrm{tot}}$ to the dust particles over the entire computational domain. As an example, assume that $\varDelta N^{e}$ number of electron macro-particles are depleted in time $\varDelta t$. The total charge $\varDelta Q$ accumulated in the dust sites over the entire computational domain is given by

24 $$\begin{aligned} \varDelta Q=ew_{e}\varDelta N^{e}, \end{aligned}$$

so that $I_{\mathrm{tot}}=\varDelta Q/\varDelta t$. The charging current for an individual dust particle can be written as

25 $$\begin{aligned} I_{d}=\frac{I_{\mathrm{tot}}}{w_{d}N^{d}}, \end{aligned}$$

from which, using OML approximation as given by Eq. (23), we can find out the average dust charge number ${\bar{z}}_{d}$ as

26 $$\begin{aligned} {\bar{z}}_{d}\simeq \left| \frac{I_{\mathrm{tot}}}{ew_{d}N^{d}}\varDelta t\right| . \end{aligned}$$

With the plasma parameters in simulation as mentioned in the beginning of the “Simulation model” section, we get a dust-charging current $I_{d}\sim -9.33\times 10^{-3}\,\mathrm{\mu A}$ at saturation of the dust charge with an average dust charge ${\bar{z}}_{d}\sim 670$. In Fig. 2, the average charging of single dust particle is shown, calculated from the total count of electron depletion in the simulation domain. As can be seen from the figure, on average, a single dust particle attains an equilibrium net charge of about $Q_{d}\sim 670e$. A theoretical estimate for $I_{d}$ can be written as^1^

27 $$\begin{aligned} I_{d}\sim 4\pi r_{d}^{2}en_{0}v_{\mathrm{th}e}\sim -4.6\times 10^{-3}\,\mu \mathrm{A}, \end{aligned}$$

which is only about half the value calculated from the simulation and can be considered to be in good agreement. According to the OML approximation^9,26^ also, the initial charging current can be written as

28 $$\begin{aligned} I_{d}=\pi r_{d}^{2}en_{0}\sqrt{\frac{8T_{e}}{\pi m_{e}}}\exp \left( \frac{e}{T_{e}}|\varDelta \phi |\right) \sim \sqrt{8\pi }r_{d}^{2}en_{0}v_{\mathrm{th}e}\sim -10^{-3}\,\mu \mathrm{A}, \end{aligned}$$

which is of the same order of magnitude as the simulation value. Note that initially the electron and ion plasma density is equal $n_{e0}\sim n_{i0}\equiv n_{0}$. In the above expression, the factor $\varDelta \phi$ is the potential difference between a dust grain and the bulk plasma, which is $\sim 0$ initially. For the parameters used in the simulation, a theoretical estimate of the initial charging time $\tau$ (till a dust particle attains an average equilibrium charge)^1^

$$\begin{aligned} \tau \simeq
\frac{\epsilon _{0}T_{e}}{n_{0}e^{2}r_{d}v_{\mathrm{th}e}}\sim 0.018\,\mathrm{\mu s}. \end{aligned}$$

which in our simulation is calculated as $\sim 0.01\,\mu \mathrm{s}$.

**Figure 3:**
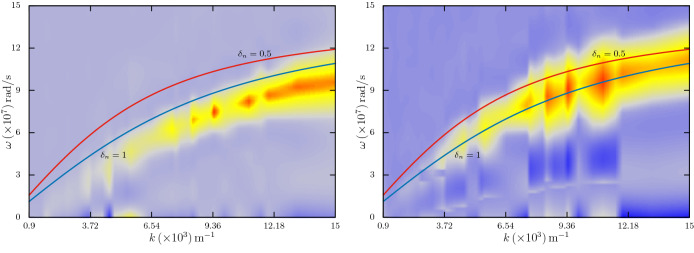
The simulated $\omega$-*k* spectra shown alongside the theoretical curves. The solid curves are same in both the panels, representing $\delta _{n}=1$ (blue) and 0.5 (red). The corresponding spectra are shown in the left and right panels, respectively. (Generated using Gnuplot, Version 5.2 patchlevel 7, URL : http://www.gnuplot.info).

### Constant dust charge

We now present the simulation results for the effect of constant dust charge on ion acoustic wave (or dust-ion-acoustic wave). It is well known that the presence of negatively charged dust particles causes the phase velocity $(\omega /k)$ of the ion acoustic wave to increase which is also associated with the Landau damping of the wave becoming less severe due to the dust particles. A linear dispersion relation of the dust-ion-acoustic (DIA) wave can be derived from the fluid theory as^9,27^

29 $$\begin{aligned} 1+\frac{1}{k^{2}\lambda _{De}^{2}}-\frac{\omega _{pi}^{2} +\omega _{pd}^{2}}{\omega ^{2}}=0, \end{aligned}$$

where $\omega _{pd}$ is the dust plasma frequency. For stationary dust particles, this relation reduces to

30 $$\begin{aligned} \omega ^{2}=\frac{k^{2}c_{s}^{2}}{1+k^{2}\lambda _{De}^{2}}, \end{aligned}$$

which can be further simplified for $k^{2}\lambda _{De}^{2}\ll 1$ as

31 $$\begin{aligned} \omega \simeq k\left( \frac{T_{e}}{m_{i}}\right) ^{1/2}\delta _{n}^{-1/2}. \end{aligned}$$

For $\delta _{n}=0.5$, with our given parameters, while Eq. (30) predicts an increase of the phase velocity by an increase of about $\sim 1.23$ as compared to the phase velocity when there are no dust particles for $k\sim 9425\,\mathrm{m}^{-1}$, for Eq. (31) the value is $\sim 1.41$. For varying *k*, we need to solve the ion acoustic dispersion relation numerically as given by Eq. (10). Our choice of *k* corresponds to mode 6 of perturbation in the periodic domain, $k=2\pi n/L$ with $n=6$.

**Figure 4:**
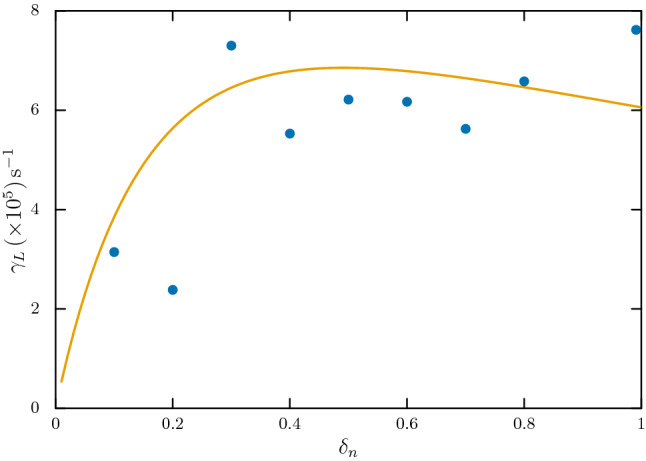
Linear Landau damping rate $\gamma _{L}$ for an *e*-*i* plasma with constant dust charge. (Generated using Gnuplot, Version 5.2 patchlevel 7, URL : http://www.gnuplot.info).

**Figure 5:**
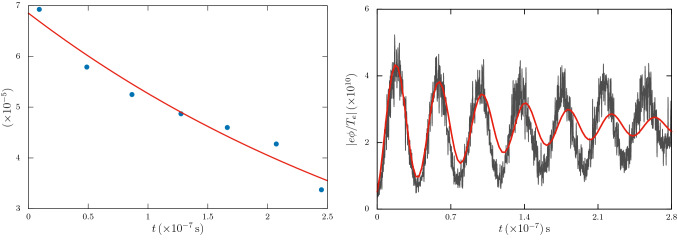
The damping of the ion acoustic wave due to dust charge fluctuation. The panel on the left shows the amplitude of the ion acoustic wave with time fitted with the best fit damping rate $\gamma _{D}\sim 2\times 10^{6}\,\mathrm{s}^{-1}$. The panel on the right shows the actual simulation data, superimposed with the theoretically calculated damping curve with $\gamma _{D}\sim 4\times 10^{6}\,\mathrm{s}^{-1}$. (Generated using Gnuplot, Version 5.2 patchlevel 7, URL : http://www.gnuplot.info).

In Fig. 3, we show this effect as simulated through our dust charging model. Both the panels in the figure show the theoretically calculated $\omega$-*k* curves from solutions of Eq. (10) for $\delta _{n}=1$ (blue colour, no dust) and 0.5 (red colour, fifty percent depletion of electrons on dust particles) without any dust charge fluctuation. Superimposed in both the panels, are the reconstructed $\omega$-*k* spectra through the simulation—for $\delta _{n}=1$ (left panel) and $\delta _{n}=0.5$ (right panel). As we can see, both the simulated spectra closely follow the theoretical curves. The associated effect corresponding to this increase of phase velocity is the overall decrease of severity of Landau damping of the ion acoustic wave^9,28–30^. There are several experimental works^28,29^ which investigated this effect in the past. We note that though the parameter regimes over which these experimental works are carried out favours decrease of the Landau damping rate $\gamma _{L}$, an actual numerical evaluation of the same via Eq. (16) may also show increase of the damping rate^31^, depending on the values of $\delta _{n}$ and *k*. We also note here that in absence of any dust dynamics, the effect of dust charge on Landau damping of ion acoustic wave, as per Eq. (16), is limited only to the value of $\delta _{n}$. In left panel of Fig. 4, we show the estimated Landau damping rate $\gamma _{L}$ from simulation as one varies $\delta _{n}$ from 0 to 1. The solid line in the figure represents the theoretical estimate. We find that the simulation results on average mimic the theoretical values. The whole computation takes about 1 h in a 4-core CPU machine for $t\simeq 20\tau _{\mathrm{ia}}$.

### Dust charge fluctuation

The dust charge fluctuates due to varying electron and ion currents to the dust particles. This charge fluctuation primarily leads to a noticeable damping of the ion acoustic wave^9,10,12^. As discussed above, even without dust charge fluctuation, the ion acoustic wave suffers Landau damping, which is shown in Fig. 4. For the plasma parameters used in this simulation and for $1>\delta _{n}>0.4$, the Landau damping rate $\gamma _{L}\simeq 6\times 10^{5}\,\mathrm{s}^{-1}$ (see Fig. 4). The additional linear damping due to dust charge fluctuation can be calculated numerically from the full dispersion relation Eq. (10), which for the simulation parameters, comes out to be $\gamma _{D}\sim 4\times 10^{6}\,\mathrm{s}^{-1}$. This effect is very clearly visible as the damping rate of the ion acoustic wave in presence of dust charge fluctuation is almost one order of magnitude larger than the damping when the fluctuation was absent for the same dust charge ${\bar{z}}_{d}$.

In the left panel of Fig. 5, the dots represent the average amplitude of the perturbation and the solid line is the best fit line of the linear damping rate $e^{-\gamma _{D}t}$, which is estimated to be about $\gamma _{D}\sim 2\times 10^{6}\,\mathrm{s}^{-1}$, which is quite close to the theoretically estimated value. The panel on the right in Fig. 5 shows the actual simulation data, superimposed with the theoretically calculated damping oscillations (solid red colour line). In the simulation, we find out the damping rate from the electrostatic oscillations, measured in terms of the square root of potential energy $|e\phi |^{1/2}$.

## Conclusions

To conclude, we have investigated the effect of dust charge fluctuation on ion acoustic wave using a 1-D hybrid-PIC-MCC algorithm. Our model is able to predict the damping of ion acoustic wave due to dust charge fluctuation, with the damping rate agreeing quite well with the corresponding theoretical estimate, which is demonstrated for the first time in this work, using a PIC code. This damping is a result of the coupling between the dust charge fluctuation and the ion acoustic wave through the plasma density variations. Our numerical model could reproduce all the benchmark results for ion acoustic wave in presence of negatively charged dust particles. These results include increase of phase velocity of ion acoustic wave in presence of negatively charged dust particles as well as decrease in Landau damping rate of the wave as dust charge increases. All these results agree quite well with the corresponding theoretical estimates. On a future outlook, this method can very well be extended for two and three dimensional geometry.

Nevertheless, we would like to emphasise that our numerical scheme does not represent the dust charging dynamics in details as far a single dust particle is concerned but it does result compatible effects of the dust charging dynamics averaged over the computational cell. This compromise leads to a considerable speed up in the calculation while capturing the effect of the dust charge dynamics on ion acoustic wave. Finally, we would also like to mention that we have used this particular numerical concept as proof of principle to demonstrate its efficacy. Though we have used a number of simplified assumptions in respect of dust charging dynamics, these assumptions can very well be relaxed and the scheme should still work. For example the constancy of ion density $n_{i}$ during dust charging, can be relaxed and the same method which we have adopted for electron-dust collision, can also be applied for ion-dust collision. We have taken care to see that our simulation parameters represent a typical laboratory dusty plasma, which can easily be used to study other dusty plasma environments such as space and astrophysical plasma as well.

**Figure 6:**
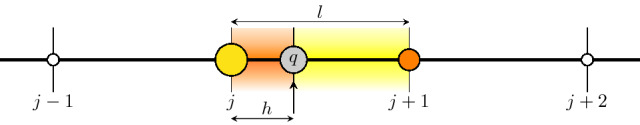
The CIC charge interpolation scheme. (Generated using PGF/Ti*k*Z, Version 3.1.4a, URL : https://github.com/pgf-tikz/pgf).

## Figures and Tables

**Figure 7:**
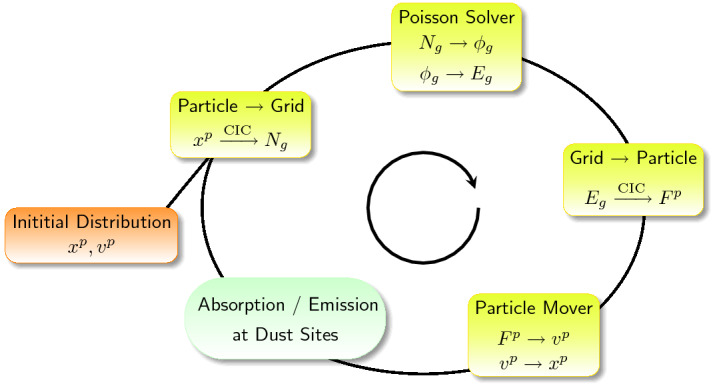
The computational cycle. (Generated using PGF/Ti*k*Z, Version 3.1.4a, URL : https://github.com/pgf-tikz/pgf).

**Figure 8:**
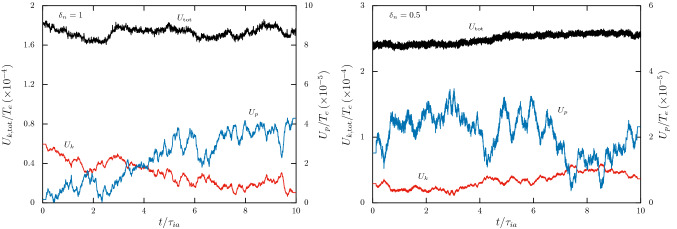
The time history of electrostatic potential energy $(U_{p})$, kinetic energy $(U_{k})$, and total energy $(U_{p}+U_{k})$ with $(\delta _{n}<1)$ and without $(\delta _{n}=1)$ dust particles, in absence of any perturbation and dust charge fluctuation. All the energies are normalised by the electron thermal energy $T_{e}$ and the time is measured in the unit of ion acoustic time scale $\tau _{\mathrm{ia}}=\omega _{\mathrm{ia}}^{-1}$. (Generated using Gnuplot, Version 5.2 patchlevel 7, URL : http://www.gnuplot.info).

## Appendix: Computational details

For the sake of clarity and completeness, in this “Appendix”, we very briefly describe our hybrid-particle-in-cell (PIC)-Monte Carlo Collision (h-PIC-MCC) electrostatic simulation model, that we have used to investigate the physical problem. For a detailed description of the PIC model, the reader can refer to some excellent monographs and review articles available elsewhere^25,32^. The following description is also detailed in one of our earlier papers^33^.

It is well known that the PIC algorithm uses computational macro-particles (also known as super-particles) instead of real particles, so that the distribution function $f_{s}$ for each species is a superposition of several computational macro-particles^33–35^,

32 $$\begin{aligned} f_{s}(x,v,t)=\sum _{p}f^{p}(x,v,t), \end{aligned}$$

where the superscript *p* denotes the attribute of the computational particles. The particle distribution function $f^{p}$ can be defined as,

33 $$\begin{aligned} f^{p}(x,v,t)=N^{p}S_{x}(x-x^{p})S_{v}(v-v^{p}), \end{aligned}$$

where $S_{x,v}$ are the shape functions of the computational particles and $N^{p}$ is the number of computational particles. In our case $S_{v}(v-v^{p})=\delta (v-v^{p})$, so that all the physical particles within a computational particle have the same velocity. Besides $S_{x}(x-x^{p})=\delta (x-x^{p})$, which means that they are point particles. It should be noted that the shape functions must satisfy the unitarity condition^25,34^

34 $$\begin{aligned} \int _{-\infty }^{+\infty }S_{\xi }(\xi -\xi ^{p})\,d\xi =1, \end{aligned}$$

where $\xi$ stands for any phase space coordinate. As all the dynamics are treated in terms of the computational particles, the Vlasov equation for species ‘*s*’ in 1-D can be written as,

35 $$\begin{aligned} \frac{\partial f^{p}}{\partial t}+v\frac{\partial f^{p}}{\partial x}+\frac{q_{s}}{m_{s}}E\frac{\partial f^{p}}{\partial v}=0. \end{aligned}$$

The equations of motions for the macro-particles can be obtained by taking the zeroth order, first order moments with respect to *x*, and first order moment with respect to *v* in the phase space of Eq. (35),

36 $$\begin{aligned} \frac{dN^{p}}{dt}= & {} 0, \end{aligned}$$

37 $$\begin{aligned} \frac{dx^{p}}{dt}= & {} v^{p}, \end{aligned}$$

38 $$\begin{aligned} \frac{dv^{p}}{dt}= & {} \frac{q_{s}}{m_{s}}E^{p}, \end{aligned}$$

where $$\left\langle f^{p}\right\rangle =N^{p}$$, $$\left\langle f^{p}x\right\rangle =N^{p}x^{p}$$, $$\left\langle f^{p}v\right\rangle =N^{p}v^{p}$$, and

39 $$\begin{aligned} E^{p}=\int S_{v}(v-v^{p})\,dv\int S_{x}(x-x^{p})E(x)\,dx, \end{aligned}$$

where $\left\langle \right\rangle \equiv \int dv\int dx$. The charge density for each species ‘*s*’ is given by,

40 $$\begin{aligned} \rho _{s}(x,t)=\sum _{p}q_{s}N^{p}S_{x}(x-x^{p}). \end{aligned}$$

In the above equations, the subscript ‘*s*’ refers to the electrons $(s=e)$, ions $(s=i)$, and dust $(s=d)$. The Poisson equation is given by,

41 $$\begin{aligned} \epsilon _{0}\frac{d^{2}\phi }{dx^{2}}=-\rho =-\sum _{s=i,e,d}\rho _{s}, \end{aligned}$$

where $\phi$ is the electrostatic potential. As mentioned in “Simulation results” section, the quasi-neutrality of the plasma is maintained by demanding (assuming negatively charged dust particles) at every time step,

42 $$\begin{aligned} N^{i}w_{i}=N^{e}(t)w_{e}+{\bar{z}}_{d}(t)N^{d}w_{d}, \end{aligned}$$

where ${\bar{z}}_{d}(t)$ is the instantaneous dust charge number averaged over all the $N^{d}$ computational particles. The weights $w_{s}=L(n_{s0}/N^{s})$ for each species, where *L* is the simulation domain length, $n_{s0}$ is the initial physical density for the species ‘*s*’ (at the beginning of the simulation), and $N^{s}(t)$ is the total number of computational particle for the species ‘*s*’ at any instant of time *t*. As mentioned before, the weight $w_{s}$ is a dimensionless quantity which measures number of physical (actual) particles of a species that are being represented in a single macro-particle of the species. So, $N^{s}(t)w_{s}$ is the total number of actual physical particles present in the simulation domain at a time *t*. Note that as per our scheme, $N^{i}(t)\equiv N^{i}=\mathrm{const}.$ (see “Dust charging with PIC simulation” section).

It is also worth nothing that as this is a 1-D simulation domain, the actual volume of the simulation box is *L* which can be thought to be equivalent to a three dimensional box of length *L* with a unit breadth and width—$L\equiv L\times 1\times 1$.

### Charge interpolation

We adopt the cloud-in-cell (CIC) linear weighting scheme in order to interpolate the charge from the particle positions on to the grid points^23,33^. A schematic representation of the scheme is shown in Fig. 6, where the 1-D grid points are marked as $j-1,j,j+1,j+2$ and the macro-particle with charge *q* is between the *j*th and $(j+1)$th grid points, marked by the arrow. The assignment of charge onto the *j*th and $(j+1)$th grid points are simply the fraction of charge *q* weighted by fraction of the length from the particle to the $(j+1)$th and *j*th grid points,

43 $$\begin{aligned} q_{j}=q(l-h),\quad q_{j+1}=qh, \end{aligned}$$

where *l* is the length of the cell and *h* is the distance of the charged particle from the *j*th grid point.

### Discretisation

The full computational cycle is schematically shown in Fig. 7. The cycle begins by distributing the macro-particles with their charge and respective velocities. This is also the step where any kind of perturbation is launched. In our case, we have given a velocity perturbation to the ions which is of the form a sinusoidal wave with amplitude $\sim v_{\mathrm{th}i}$, which ensures the linearity of the perturbation. The accumulation of charges from each cell is used to calculate the charge density $\rho _{j}$ from the neighbouring particle positions through a standard interpolation function $\mathcal{F}_{\mathrm{CIC}}$ as described above,

44 $$\begin{aligned} \rho _{j}=\sum _{s,p}\frac{q_{s}^{p}}{\varDelta x}\mathcal{F}_{\mathrm{CIC}}\left( x_{j}-x^{p}\right) , \end{aligned}$$

where the summation extends over the particles in the neighbouring cells and total number of different species. The Poisson equation then can be discretized as,

45 $$\begin{aligned} \epsilon _{0}\frac{\phi _{j+1}-2\phi _{j}+\phi _{j-1}}{\varDelta x^{2}}=-\rho _{j}, \end{aligned}$$

where the subscript ‘*j*’ of the field quantities denotes the value at the middle of the computational cell. Apparently, the cell vertices are at $x_{j\pm 1/2}$. The Poisson equation is solved with the help of a successive-over-relaxation method, which provides the electric field, defined as,

46 $$\begin{aligned} E_{j}=-\frac{\phi _{j+1}-\phi _{j-1}}{\varDelta x}. \end{aligned}$$

The equivalent force is implemented in the equations of motion is through a leap-frog scheme,

47 $$\begin{aligned} x^{p,n+1}= & {} x^{p,n}+\varDelta t\,v^{p,n+1/2}, \end{aligned}$$

48 $$\begin{aligned} v^{p,n+3/2}= & {} v^{p,n+1/2}+\varDelta t\frac{q_{s}}{m_{s}}E^{p}(x^{p,n}), \end{aligned}$$

which provides the new positions and velocities for the macro-particles. However before the next computational cycle begins, the absorption (or emission) of the electrons at the dust sites is determined for each maro-particle depending upon their closest approach to the dust sites (as already described).

### Benchmarking

The benchmarking results of the code can be seen in Fig. 8, where the electrostatic potential, kinetic, and total energy, evaluated as the average energy (see below) per particle, plotted against time in absence of any external perturbation. All the initial conditions are same as described before. The left panel of Fig. 8 shows the energy history of a pure *e*-*i* plasma without any dust, while the right panel for dusty plasma where $50\%$ of the electrons are depleted in the dust particles. The quantity $\delta _{n}$ is the ratio of equilibrium electron density to the equilibrium ion density, $\delta _{n}=n_{e0}/n_{i0}$. The dust case is without any dust charge fluctuation.

The energies plotted in the figure are the average energies of a particle,

49 $$\begin{aligned} U_{p}= & {} \left\langle \sum _{N^{e},N^{i},N^{d}}\left| Q^{j}\phi \right| \right\rangle _{N}, \end{aligned}$$

50 $$\begin{aligned} U_{k}= & {} \left\langle \sum _{N^{e},N^{i},N^{d}}\frac{1}{2}M^{j}(v^{j})^{2}\right\rangle _{N}, \end{aligned}$$

51 $$\begin{aligned} U_{\mathrm{tot}}= & {} U_{p}+U_{k}, \end{aligned}$$

where the summation is over all the computational particles (macro-particles) denoted by $N^{j}$, $j=e,i,d$ for ions, electrons, and dust particles. The quantity $Q^{j},M^{j}$ are the respective charge and mass of the macro-particles. The <> denotes the average over all the computational particles $N=\sum _{j=e,i,d}N^{j}$. As one can see that the energy fluctuations which are measured in terms of electron thermal energy can very well be termed as random fluctuations, as desired.
